# Enhancing Coffee Agroforestry Systems Suitability Using Geospatial Analysis and Sentinel Satellite Data in Gedeo Zone, Ethiopia

**DOI:** 10.3390/s24196287

**Published:** 2024-09-28

**Authors:** Wondifraw Nigussie, Husam Al-Najjar, Wanchang Zhang, Eshetu Yirsaw, Worku Nega, Zhijie Zhang, Bahareh Kalantar

**Affiliations:** 1 Department of Land Administration and Surveying, Injibara University, Injibara P.O. Box 40, Ethiopia; wondifraw.nigussie@inu.edu.et; 2 Department of Land Administration and Surveying, Dilla University, Dilla P.O. Box 419, Ethiopia; 3 School of Computer Science, Faculty of Engineering and IT, University of Technology Sydney, Sydney, NSW 2007, Australia; husam.al-najjar@uts.edu.au; 4 Key Laboratory of Digital Earth Science, Aerospace Information Research Institute, Chinese Academy of Sciences, Beijing 100094, China; zhangwc@radi.ac.cn; 5 Department of Natural Resource Management, Dilla University, Dilla P.O. Box 419, Ethiopia; eshetu.yirsaw@du.edu.et; 6 Institute of Land Administration, Debre Markos University, Debre Markos P.O. Box 269, Ethiopia; worku_nega@dmu.edu.et; 7 School of Geography, Development and Environment, University of Arizona, Tucson, AZ 85721, USA; zhijiezhang@arizona.edu; 8 RIKEN Center for Advanced Intelligence Project, Disaster Resilience Science Team, Tokyo 103-0027, Japan

**Keywords:** coffee plantation, sentinel, land suitability, GIS, AHP, Gedeo

## Abstract

The Gedeo zone agroforestry systems are the main source of Ethiopia’s coffee beans. However, land-use and suitability analyses are not well documented due to complex topography, heterogeneous agroforestry, and lack of information. This research aimed to map the coffee coverage and identify land suitability for coffee plantations using remote sensing, Geographic Information Systems (GIS), and the Analytical Hierarchy Process (AHP) in the Gedeo zone, Southern Ethiopia. Remote sensing classifiers often confuse agroforestry and plantations like coffee cover with forest cover because of their similar spectral signatures. Mapping shaded coffee in Gedeo agroforestry using optical or multispectral remote sensing is challenging. To address this, the study identified and mapped coffee coverage from Sentinel-1 data with a decibel (dB) value matched to actual coffee coverage. The actual field data were overlaid on Sentinel-1, which was used to extract the raster value. Pre-processing, classification, standardization, and reclassification of thematic layers were performed to find potential areas for coffee plantation. Hierarchy levels of the main criteria were formed based on climatological, edaphological, physiographic, and socioeconomic factors. These criteria were divided into 14 sub-criteria, reclassified based on their impact on coffee growing, with their relative weights derived using AHP. From the total study area of 1356.2 km^2^, the mapped coffee coverage is 583 km^2^. The outcome of the final computed factor weight indicated that average annual temperature and mean annual rainfall are the primary factors, followed by annual mean maximum temperature, elevation, annual mean minimum temperature, soil pH, Land Use/Land Cover (LULC), soil texture, Cation Exchange Capacity (CEC), slope, Soil Organic Matter (SOM), aspect, distance to roads, and distance to water, respectively. The identified coffee plantation potential land suitability reveals unsuitable (413 km^2^), sub-suitable (596.1 km^2^), and suitable (347.1 km^2^) areas. This study provides comprehensive spatial details for Ethiopian cultivators, government officials, and agricultural extension specialists to select optimal coffee farming locations, enhancing food security and economic prosperity.

## 1. Introduction

Coffee is a significant traded commodity worldwide [1]. Coffee trees were originally identified in the Ethiopian mountains in the ninth century. Ethiopia is a country where agriculture supports more than 80% of the population [2]. To provide food for their own consumption, the majority of the population engages in subsistence farming. A small portion of the population grows cash crops, while others cultivate food crops on a large scale [2]. It is acknowledged that agriculture accounts for a large portion of foreign exchange earnings. This is evident from the cash crops grown throughout the nation [3]. Coffee, tea, and other fruits are the principal cash crops grown in Ethiopia, with coffee currently being the most valuable. Ethiopia is the world’s fifth-largest producer of coffee after Indonesia and exports its crop to other countries [4].

Estimates showed that the Gedeo zone has the potential for 67,164 ha of land to produce coffee, of which 51,698.35 ha do so on an annual basis, and provides a living for 644,000 people [5]. Since 2023, the Gedeo Cultural Landscape has been recognized as a UNESCO World Heritage Place. The landscape has heterogeneous agroforestry and diverse topography. The zone is mainly known for its coffee agroforestry system, particularly by the Yirgachefe brand. Yirgachefe Coffee is an export brand of coffee in Ethiopia. However, the existing coffee land use is not well mapped, making it difficult to easily detect where this land-use system is located in different districts of the study region. Hence, the lack of successful, broad coffee extent maps has hampered a comprehensive awareness of coffee’s overall environmental effect [6].

In agroforestry systems (AFS), coffee plants flourish in the understory of indigenous or introduced tree species [7]. Among the factors impeding precise AFS identification for coffee are shade tree coverage, topographic variation, and AFS structural complexity [8]. The widespread presence of alien tree plantations in indigenous forests makes it difficult to identify and map understory coffee using satellite imagery [8]. In recent studies, it has been possible to recognize and distinguish between different types of land use by combining Synthetic Aperture Radar (SAR) with multi-temporal optical reflectance images [9]. With optical and SAR sensors on board, the Sentinel mission offers the chance to combine optical and radar data to improve mapping capability on overcast days [10]. The European Space Agency’s Copernicus Sentinel-1 mission can monitor the earth’s surface using radar with high spatial and temporal resolution [10,11]. SAR provides observations with a ten-meter spatial resolution for crop monitoring [12]. Sentinel-1A’s backscattering coefficient is used to calculate the types of heterogeneous features that exist on the surface of the land. For instance, the computed moisture content of soil from the SAR image using relative decibel values indicated the effectiveness of SAR in soil and hydrological applications [13,14]. On the other hand, Sentinel-2A’s Multispectral Instrument (MSI), primarily utilized for LULC monitoring, has a higher spectral and spatial resolution [15].

The current requirement for optimum land use is greater than ever before due to rapid population growth and urban sprawl, which have made land a relatively limited commodity for farming and other uses. As a result, there is a rising urgency to match land capacities and land uses in the most reasonable way [16,17]. Remote sensing and Geographic Information Systems (GIS) technologies are necessary to detect the most suitable sites for sustainable agricultural production in accordance with their environmental potential [18]. Selection of the best site for agricultural production requires consideration of technical needs as well as physical, financial, social, and ecological factors that could resolve conflicts of interest by using several decision-support approaches [19]. Various efforts have been made to use GIS in conjunction with multi-criteria decision-making techniques to select suitable sites [20]. Incorporating multi-criteria decision analysis methods into GIS has resulted in robust support for spatial decision-making systems, making it possible to quickly develop maps of land suitability.

According to [21,22], climate-related factors mostly affect the environmental suitability of Arabica coffee plantations. When assessing Arabica coffee’s ecological compatibility, the MaxEnt and Analytical Hierarchy Process (AHP)-GIS approaches have proven reliable. In the past few decades, many land suitability assessment issues [18,23,24,25,26,27,28] have been addressed using AHP and GIS-based land suitability analysis for various types of land uses. On the other hand, no documentation is currently available regarding the degree of deforestation in Ethiopia’s coffee-growing regions, and no successful mapping of these coffee forests has been accomplished [29]. Thus, it is evident that no appropriate mapping techniques have been created for the Ethiopian coffee forests. Moreover, there is little available data on the dispersion of coffee plantations [1]. Hwang et al. [29] investigated the effects of intensive management of coffee trees on ecological diversity and regeneration using prospective satellite-based mapping, field photography, and tree survey methodologies. Moreover, several techniques for mapping coffee plantations were previously used by researchers. These include pixel-based classification [30], object-based classification [31], and hybrid classification [32]. However, these studies have limitations in mapping exact coverage due to spectral mixing. Thus, it is challenging to develop universal mapping techniques due to the unique characteristics of each research area and the agroforestry strategies used in each location [33]. Though obtaining the spatial distribution of coffee farmland and finding its land suitability are crucial for long-term coffee management [1], the previous studies mentioned above did not consider the edaphological thematic factors for Ethiopian coffee plantation suitability. Additionally, the mapping methodology using Landsat images has limitations due to the spectral similarity of the canopy tree coverage. Thus, to overcome the previous research limitations, the current study investigated the coffee farm suitability in Gedeo agroforestry by considering the soil (edaphological) properties, such as soil texture, soil organic matter, soil pH, and soil cation exchange capacity, in conjunction with metrological, environmental, and socioeconomic thematic factors. In addition, the spatial coffee coverage was inspected by using the ground truth data and sentinel SAR decibel (dB) value, which is not affected by spectral mixing.The 2021 LULC of the heterogeneous land use in the Gedeo zone has been evaluated using the Sentinel 2-A data. In comparison to pixel-based picture categorization, object-based mapping algorithms have been shown to be effective at a variety of scales in the literature [34,35].

Observations on coffee productivity and plant stress, as well as responses from coffee-growing communities, suggest that the effects of climate change are already having an adverse impact [36]. Throughout the next few decades, Ethiopia’s coffee crop will continue to be influenced and altered by climate change [21]. Accordingly, in the future, many areas that are currently ideal for growing coffee will lose some of their suitability and, in certain circumstances, become unsuitable. Conversely, a significant portion of land that was previously unsuitable for coffee production will become suitable. Additionally, throughout this century, several higher altitude regions will become more conducive to coffee production. Resilience in the Ethiopian coffee industry will depend heavily on migration to these regions. To this end, this research aimed to map coffee agroforestry and identify land suitability for potential coffee-growing areas using integrated Sentinel satellites, GIS, and the AHP method in the Gedeo zone of Ethiopia.

## 2. Materials and Methods

### 2.1. Description of the Research Area

**Figure 1:**
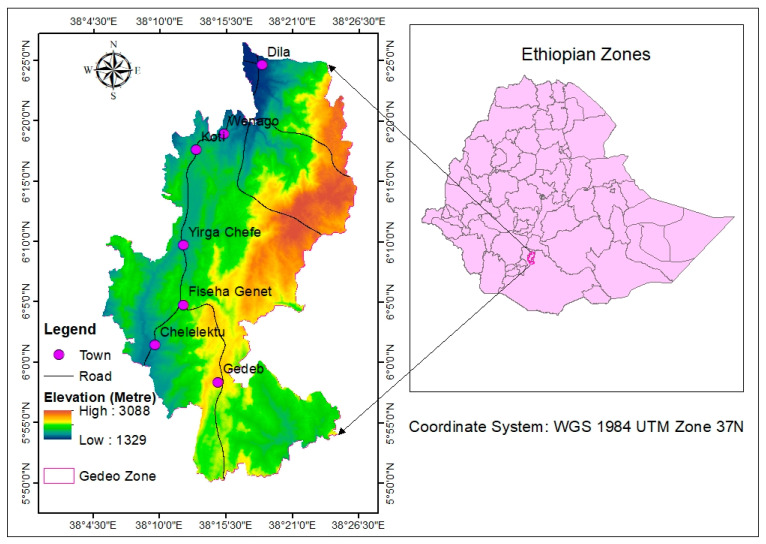
Location of the study area.

The study area covers the entire Gedeo zone of the SNNPR region (Figure 1), which is located 390 km far from Addis Ababa, the capital of Ethiopia. The overall study area covers 1356.2 km^2^ or 135,622.3 ha, which is bounded by longitudes of 38°05′13″ to 38°26′19″ E and latitudes of 5°50′8″ to 6°26′31″ N. The research region lies between 1329 and 3088 m above mean sea level, including a slight gradient to extremely sloping. The agro-ecological region of the study area is classified as Dega, Woyina Dega, and Kolla [37].

The mean monthly minimum and maximum temperatures are 18 °C and 25 °C, respectively. According to [38], the mean yearly temperature in Gedeo zone is 20.6 °C, which is ideal for growing coffee plantations. In similar study, majority of cultivators (87.8%) in the Gedeo zone relied on rainfall because the region receives approximately 1129 mm of precipitation annually, which is the ideal amount needed by coffee for healthy growth and yield. However, 12.2% of the farms were using rivers and rainwater collection for additional irrigation. In Ethiopia, the Gedeo zone is known as a highly densely populated area [39]. According to [37], approximately 1,541,522 people are living in the Gedeo zone overall, with 1,159,602 living in rural areas and 381,920 in urban areas. With the famous specialty coffee brand “Yirgacheffe coffee” Gedeo is currently one of Ethiopia’s main coffee-producing regions. Coffee cultivation serves as the main source of income for the local population.

### 2.2. Data and Software

This research used different data and software to map LULC, spatial coffee coverage, and to identify land suitability for coffee growing. ArcGIS software is the product of the Environmental Systems Research Institute (ESRI) (headquartered in Redlands, CA, USA), which is a complete geospatial platform mainly containing ArcCatalog, ArcMap, and Arctoolbox. Those components are used to manage, display, retrieve, analysis, and present geospatial data for effective decision-making. A raster-based software program designed especially for processing satellite images and extracting data is called ERDAS Imagine. IMPACT-Toolbox is a user-friendly toolbox mainly used for multispectral image processing. SNAP is a product of the European Space Agency, which is an open-source Sentinel Application Platform, designed for sentinel image processing. The eCognition software is mainly focused on image segmentation and classification processes. For the current study, ArcGIS 10.5 was used for map preparation. ERDAS Imagine 2015, IMPACT-Toolbox 5.1, and SNAP 7.0 were used for image processing. The eCognition Developer 9.01 was used for image classification. Different types of data were gathered and organized for analysis using both primary and secondary data sources (Table 1). ALOS PALSAR digital elevation model (DEM) data with a spatial resolution of 12.5 × 12.5 m from Alaska facility wer employed to extract elevation, terrain slope, terrain aspect, and distance to water network. The 30 years (1991–2021) of temperature information such as annual mean maximum temperature, annual mean minimum temperature, and average annual temperature were gathered from Ethiopia’s National Meteorological Agency. Climate Hazards Group InfraRed Precipitation with Station (CHIRPS) provided the 30 years of mean annual rainfall data (1991–2021). Recently, a gridded Soil Information System of Africa at 250 m resolution was created by the Africa Soil Information Services (Wageningen, The Netherlands) (https://soilgrids.org) project to display the spatial distribution of primary soil properties that are relatively stable. These properties include the depth to bedrock, the texture of the soil, pH, and the contents of coarse fragments, organic carbon, and exchangeable cations like Ca, Mg, Na, K, and Al, as well as the corresponding cation exchange capacity (CEC) [40,41]. Sentinel images (sentinel-1 and 2A) and Global Positioning System (GPS) information comprise the main data. Integrated optical sentinel 2A cloud-free image and RADAR sentinel-1 were used to prepare land-use/land-cover maps.

The Sentinel-1A and Sentinel-1B SAR satellites were launched as part of the Copernicus program to survey and monitor the topography of the earth and create practical applications for studying the environment [42]. Sentinel-1 is a RADAR imaging satellite that collects images at every moment of day and in any type of weather using a C-band SAR instrument that works at a frequency of 5.405 GHz [13]. With a 10 m spatial resolution, these dual-satellite constellations provide a six-day repeat frequency for every part of the world [14]. The acquisition modes that the Sentinel-1 can use are Interferometric Wide swath (IW), Strip map (SM), Extra-Wide swath (EW), and Wave (WV). The Sentinel-1 images were dual-polarized in both vertical transmit and vertical receive (VV) and vertical transmit and horizontal receive (VH) modes [43]. This study used a Sentinel-1A SAR image that was obtained on 21 January 2021, with the Ground Range Detection (GRD) product, IW mode, VH polarization, and descending.

Sentinel-2A is a wide-swath, excellent quality, multispectral imagery mission, and aims to examine the planet’s land surfaces thoroughly and methodically. Scholars in [14,43,44,45] state that the Sentinel-2A image set consists of 13 spectral categories with spatial resolutions of 10 m, 20 m, and 60 m. Sentinel-2’s spectral bands offer information for classifying LULC [46]. For this study, cloud-free 8 January 2021 Sentinel-2A image was accessed through the Copernicus scientific data hub and used for LULC classification. Because of their resolution [13], Sentinel-2A’s bands of two, three, four, eight, eleven, and twelve were chosen for stacking.

### 2.3. Data Processing

**Figure 2:**
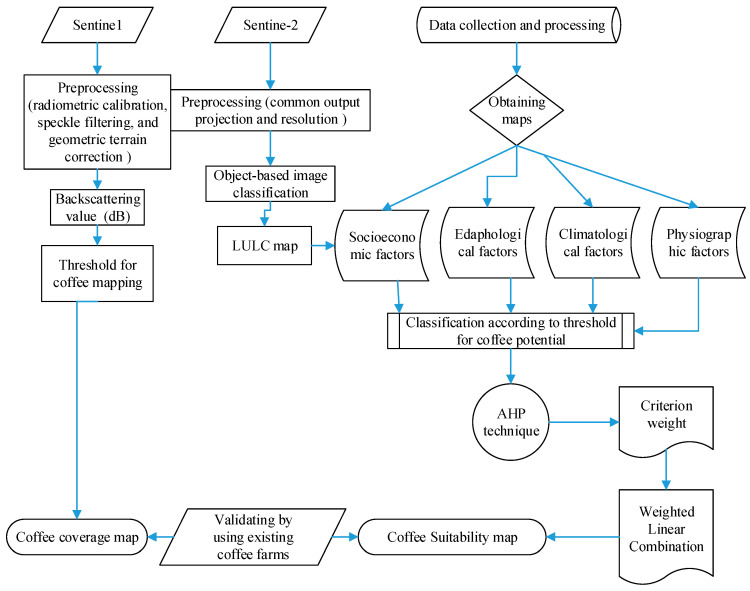
Methodological flowchart of the research.

**Figure 3:**
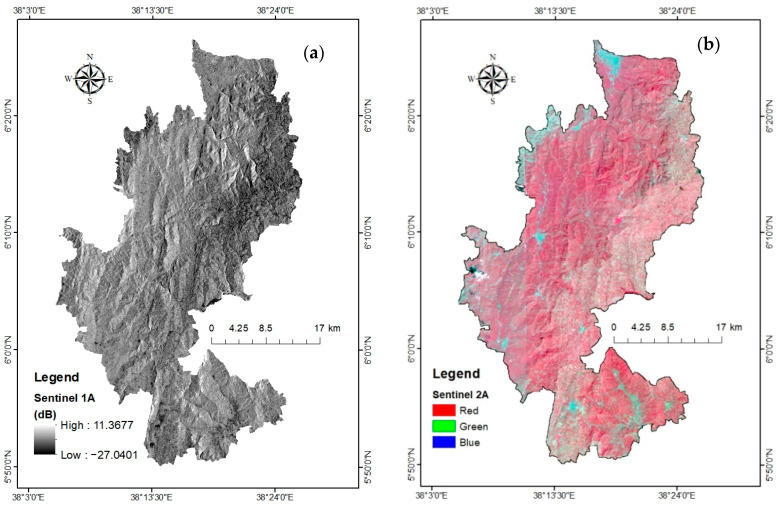
Pre-processed images (**a**) sentinel-1 (SAR), and (**b**) sentinel-2 (MSI).

In order to ensure consistency, Xiong et al. [47] employed data from reputable sources like government agencies, reputable research institutes, and satellite earth observation. The current study used satellite and GIS axillary data from known sources, which were widely applicable in prior studies. Then, data processing and standardization were performed. Figure 2 presents the overall methodological flowchart for the study. Radiometric and geometric corrections were applied on the acquired sentinel 1 and 2 images. In the sentinel 1 image, image calibration, speckle filtering, and geometric terrain correction were the pre-processed stages used for this study. These preprocessing steps were performed using the Sentinel Application Platform (SNAP 7.0) (Figure 3a). Previous works [13] explained the sentinel 1 and 2 comprehensive preprocessing methods employed in this investigation. The IMPACT-Toolbox 5.1 program is used to establish the common output projection and resolution of Sentinel-2 data. Thus, this research has used IMPACT-Toolbox program, and the closest-neighbor resampling while merging bands of different resolutions to produce an image with a maximum resolution of 10 m (Figure 3b). The Top of Atmospheric Reflectance Byte was computed. Moreover, the climatological, edaphological, physiographic, and socioeconomic factors were used on a similar coordinate system and pixel size similar to the processed sentinel images. After data processing, extensive field visit was performed, which was used to interpret remote sensing data. We interpreted the different thematic layers separately and used them to map coffee spatial coverage and to find coffee-growing suitability.

### 2.4. Agro-Ecological Classification

**Figure 4:**
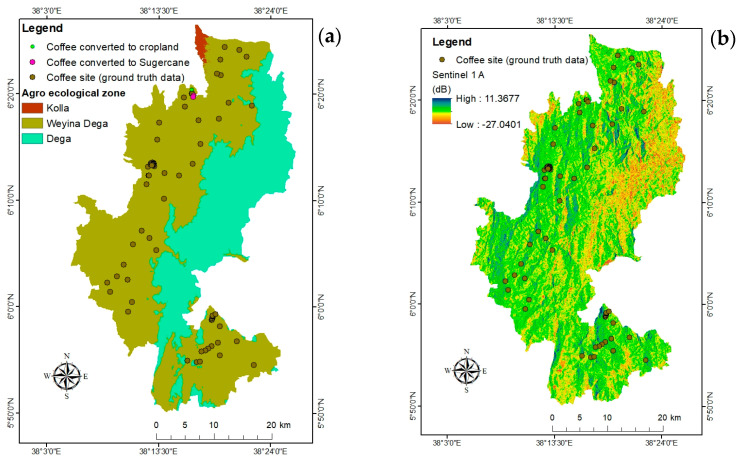
Agro-ecological classification, (**a**) sample field data of coffee coverage, and (**b**) processed sentinel-1.

Likely the most common way of arranging geographic space to comprehend current and projected crop production is the use of agro-ecological zones [48]. Agro-ecological classification divides land-use types or production systems into comparatively homogeneous units by using the biophysical characteristics of the soil, landscape, and climate. Based on customary zone classifications that are frequently used by rural communities, Hurni [49] established a set of agro-ecological zone categories for Ethiopia (Table 2). Mappable boundaries were placed on agro-ecological zones as a result of these classifications, which include precise data related to elevation and precipitation (Figure 4a).

### 2.5. Mapping of Coffee Potential Areas

Remote sensing classifiers often confuse agroforestry and plantations like coffee cover with forest cover; this is because these land-cover classes often have similar spectral signatures. The degree of difficulty and labor required to implement the coffee area mapping technique reveals its level of complexity [6]. One reason for the paucity of information regarding coffee cultivation locations and environmental effects is the difficulty of mapping coffee using satellite imagery. Using SAR data is one method of overcoming continual cloud cover. The texture (spatial variation) of SAR imaging varies in addition to its intensity. Texture, a quantitative assessment of a pixel’s association with its neighboring pixels, is frequently utilized to raise the standard of studies on land-use classification [50]. Regardless of its accuracy, most researchers have used Maximum Likelihood (ML) as a parametric algorithm to map coffee plantations [51,52]. Given the variability of coffee production systems and the complex landscape features of coffee systems, mapping coffee extent necessitates careful consideration of the remote sensing analytical options. A Sentinel-1/Sentinel-2 fusion strategy is a very effective technique [6]. The constraints posed by optical vision in overcast locations can be mitigated with the increased availability and coverage of data from SAR sensors [53]. The SAR signal is helpful for mapping coffee AFS since it can reach the lowest vegetation layers. Considering this, the current study estimated and mapped coffee coverage from Sentinel-1 data using dB values matched to actual coffee coverage. The actual field data (coffee coverage) were overlaid on processed Sentinel-1 data (Figure 4b), which were used to extract the raster value. The extracted raster values ranged from −19.034781 to −10.773612 dB and were used as a threshold for coffee mapping.

### 2.6. Preparation of Thematic Layers for Coffee Land Suitability

Preliminary processing, categorization, standardization, and reclassification of various datasets were all a part of the data preparation procedures. Based on [54], the major and subordinate factors that influence the growth of coffee plantations in Gedeo were identified. Hierarchy levels of main criteria were constructed based on climatological, edaphological, physiographic and socioeconomic factors. Those four main criteria were split into 14 sub-criteria. We took into account the following sub-criteria for climatology: mean annual rainfall, average annual temperature, mean lowest temperature and mean maximum temperature. Texture, pH, Soil Organic Matter (SOM), and CEC were the edaphological sub-criteria. The three sub-criteria of physiography used in this study were aspect, slope, and elevation of the terrain. Sub-criteria of Land Use/Land Cover (LULC), distance to the road network, and distance to the water network were the division of the Socioeconomic main criteria. The sub-criteria were rearranged and assigned weights into three categories based on the territory’s suitability standards [54,55]: “Suitable” (3), “Subsuitable” (2) and “Unsuitable” (1) (Table 3).

#### 2.6.1. Physiographic Thematic Layers

##### Elevation

**Figure 5:**
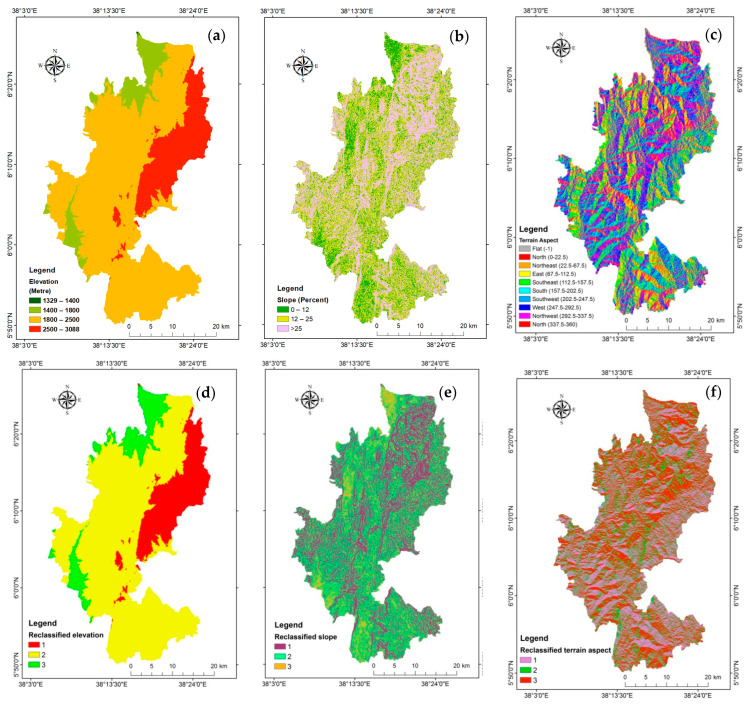
Maps of physiographic thematic layers (**a**) Elevation, (**b**) Slope, (**c**) Aspect, (**d**) Reclassified elevation, (**e**) Reclassified slope, (**f**) Reclassified aspect.

Geographical locations have proven crucial for the cultivation and production of coffee [22]. According to Zhang et al. [22], the main variable affecting the quality of Arabica coffee is altitude. High-altitude regions have a protracted coffee growth cycle due to their wide diurnal temperature fluctuations. Extreme elevation could result in a drop in temperature and rainfall, which would be adverse to the development of coffee [30]. The study region is elevated between 1329 and 3088 m above mean sea level (Figure 5a,d). The topographic complexity in the study area poses difficulties in the interpretation of remote sensing data and land suitability analysis.

##### Slope and Aspect

The land’s slope significantly influences the amount and speed of water runoff (Figure 5b,e). According to Hidayat et al. [61], a steeper slope results in increased flow speed, which in turn reduces water penetration into the ground. The rate at which nutrients are utilized, soil respiration, and soil depth are all affected by the slope [22]. Consequently, a gentle gradient is a better place to produce Coffea arabica. The temperature and duration of light are primarily affected by the slope direction [22]. The bright, semi-shaded, and semi-sunny slopes are ideal for growing Arabica coffee (Figure 5c,f).

#### 2.6.2. Edaphological Thematic Layers

**Figure 6:**
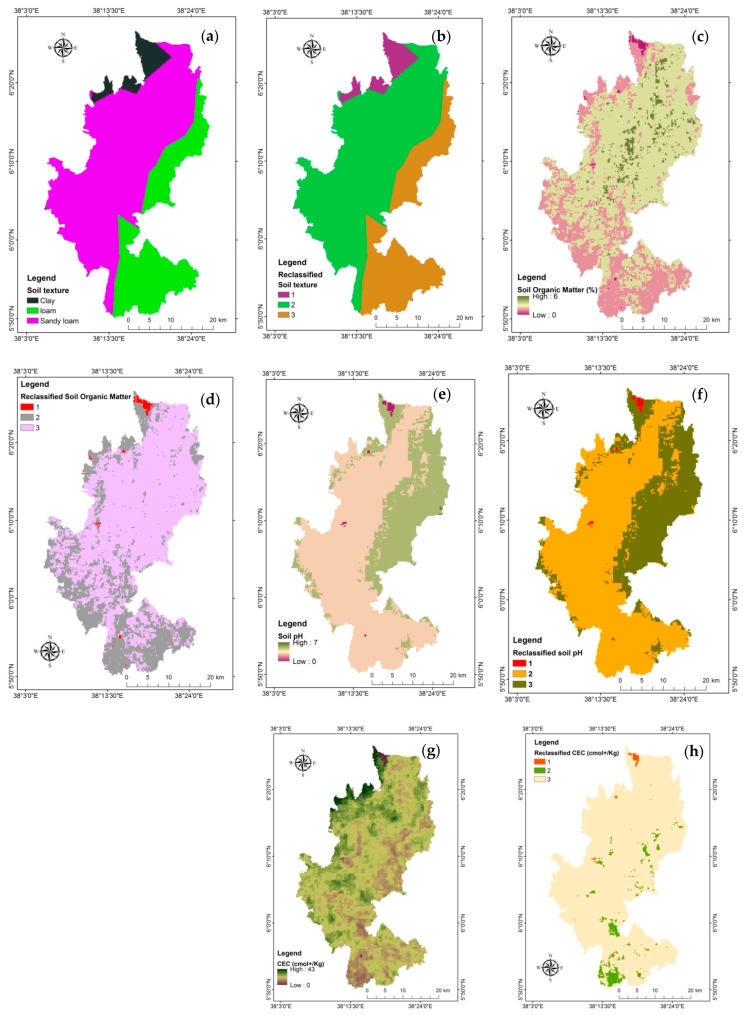
Maps of edaphological thematic layers. (**a**) Soil texture, (**b**) Reclassified soil texture, (**c**) Soil organic matter, (**d**) Reclassified soil organic matter, (**e**) Soil pH, (**f**) Reclassified soil pH, (**g**) CEC, (**h**) Reclassified CEC.

Soil properties are very important for the growth and development of coffee [22]. The edaphological thematic layers considered for the current study include soil texture, soil organic matter, soil pH, and soil cation exchange capacity (Figure 6).

According to Zhang et al. [22], soil type and soil texture mainly affect the root extension and drainage of coffee. The most suitable soil textures are sandy loam and sandy clay loam, which are loose, aerated, and permeable [62]. The types of soil texture in the Gedeo zone include clay, loam, and sandy loam (Figure 6a), with most of the area covered by sandy loam (Table 4). The reclassified soil texture for coffee plantations is presented in Figure 6b.

##### Soil Organic Matter

Soil organic matter (SOM) can be defined as entirely organic supplies found in soil that are part of or have been part of living organisms [63]. Organic C content may vary due to differences in the type and amount of vegetation on the land. The low organic content indicates the low production of organic matter [64]. The development of coffee plants and their leaves’ net photosynthetic velocity were determined by the amount of organic matter present [22]. The research area’s SOM varies from 0% to 6% (Figure 6c). The SOM is reclassified into suitable, sub-suitable, and unsuitable for coffee plantation (Figure 6d).

##### Soil PH

In both controlled and unmanaged environments, soil pH is a key variable in the field of soil science that represents the relative acidity or alkalinity of the soil [65]. Due to its master variable status and pivotal role in numerous soil processes, knowledge of the spatial variation of soil pH is essential for a wide range of stakeholders in various scientific domains [66]. The soil in Gedeo zone province was mainly acidic with a pH range of 0–7 (Figure 6e). The reclassified soil pH is depicted in Figure 6f.

##### Cation Exchange Capacity (CEC)

The CEC is the soil’s ability to retain positively charged ions. The main reason soils have a CEC is that the organic matter and clay particles in the soil have a tendency to be negatively charged. Plant nutrient availability and retention in the soil are predicted by CEC, a key soil characteristic. Cation exchange takes place on the surfaces of clay minerals, organic matter, and roots, which is why soil CEC normally rises with increasing clay content and organic matter. In comparison to acidic circumstances, soil OM will generate a higher CEC at nearly neutral pH (pH-dependent CEC). As a result, adding organic material will probably cause a soil’s CEC to gradually rise. The CEC in the study area ranges from 0 to 43 (Figure 6g) cmol+/kg, which categorized clay and clay loam texture. The majority of the CEC in the study falls into the suitable and sub-suitable categories, respectively (Figure 6h).

#### 2.6.3. Climatological Thematic Layers

##### Rainfall

**Figure 7:**
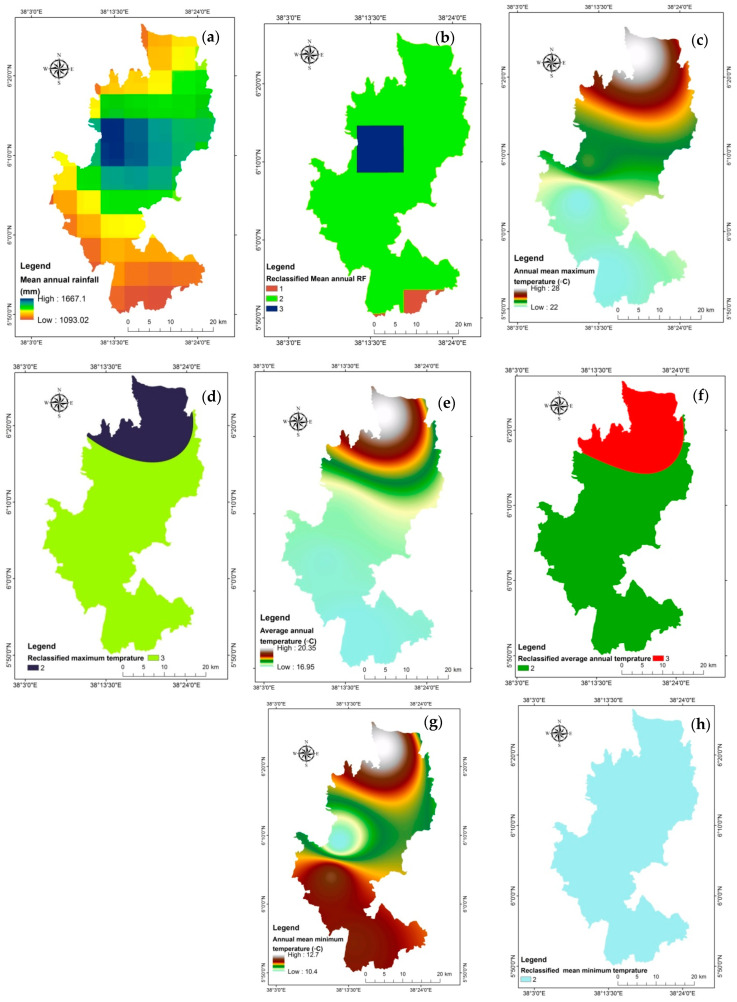
Maps of climatological thematic layers, (**a**) Mean annual rainfall, (**b**) Reclassified mean annual rainfall, (**c**) Annual mean maximum temperature, (**d**) Reclassified maximum temperature, (**e**) Average annual temperature, (**f**) Reclassified average annual temperature, (**g**) Annual mean minimum temperature, (**h**) Reclassified mean minimum temperature.

The effects of climate change on the amount and quality of coffee produced have recently attracted attention because they have not been taken into account in past studies [67]. Rainfall is crucial to the development of coffee [22]. According to Laderach et al. [68], excessive rainfall causes flower drop and fruit impairment, while insufficient rainfall causes drought and decreased yield. The 30 years (1991–2021) mean annual rainfall at Gedeo zone ranges from 1093.02 to 1667 mm (Figure 7a). Most rainfall coverage in the current research zone was a sub-suitable range for coffee plantation. Areas around Yirgacheffe are the most suitable, and edge of the southern part is unsuitable for coffee plantation (Figure 7b).

##### Temperature

One significant climatic aspect that affects how coffee plants grow and develop is temperature [61]. Arabica coffee grows well in the highlands with optimal temperatures between 150 and 240 degrees Celsius (°C) [61]. However, the coffee tree was vulnerable to agricultural climatic disasters such as extreme cold and heat [22]. The right atmosphere was one of the main elements ensuring coffee’s regular growth. When the daily low falls below 0 °C, the coffee tree will suffer freezing harm and eventually die. If the maximum daily temperature is too high, the coffee leaves will fade and wither, which will be bad for flowering and fruiting [69]. Similar to Nigussie et al. [13], the temperature maps (1991–2021) were created in ArcGIS by using the Inverse Distance Weighting (IDW) interpolation method on data from six rain gauge-stations (Table 5; Figure 7c,e,g). The average annual temperature, annual mean maximum temperature, and annual mean minimum temperature are reclassified based on coffee plantation suitability criteria (Figure 7d,f,h).

#### 2.6.4. Socioeconomic Thematic Layers

##### LULC

**Figure 8:**
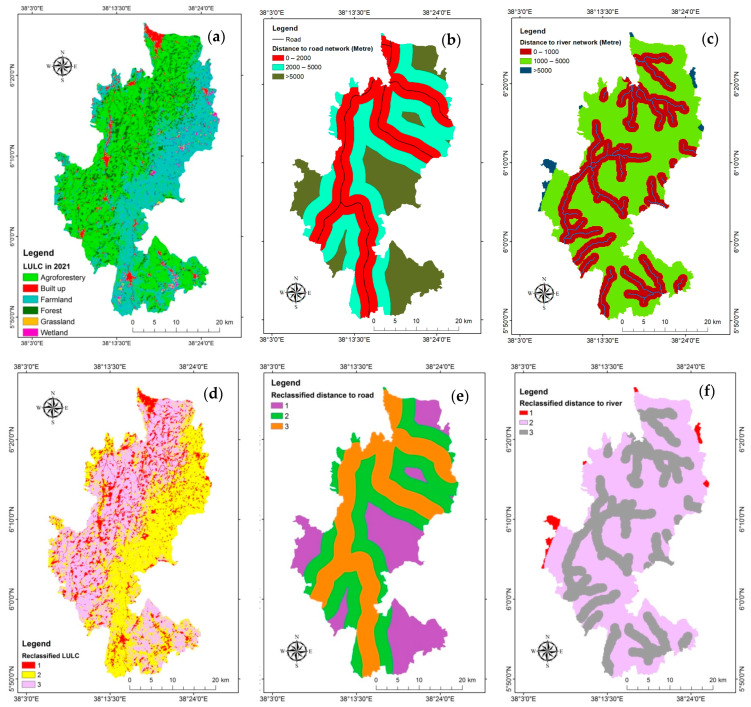
Maps of socioeconomic thematic layers, (**a**) LULC in 2021, (**b**) Distance to road network, (**c**) Distance to river network, (**d**) Reclassified LULC, (**e**) Reclassified distance to road, (**f**) Reclassified distance to river.

Land use maps are a crucial planning tool for decision-makers because they show the geographical distribution of human settlements, cultural landscapes, and natural resources [70]. The OBIA technique performs better in terms of classification accuracy for high and very high resolutions than the pixel-based technique [71]. Segmentation creates the picture objects used in the grouping process in OBIA, which is always the initial phase of any process in the eCognition development. The segmentation of image information into things at different scales is a component of the OBIA approach. Scale, form, and compactness are taken into account while segmenting the basic objects in an image [71]. Resulting from the image segmentation procedure, one of the algorithms in OBIA that is most frequently employed is multiresolution segmentation. For a specific resolution of image objects, the multi-resolution segmentation algorithm (MRS) locally reduces the average heterogeneity of picture objects. It can be put into action at the pixel level to create new picture objects on a new image object level or on an existing image object level. Based on settings like scale, color, and shape, smaller things are combined into larger objects during the process. The estimation of scale parameter (ESP) tool was utilized in this work to reduce trial and error, which is crucial for determining the scale parameter [8,72]. Currently, OBIA uses high-quality imageries like those from Sentinel-2 [73]. Using the nearest neighbor classification technique and OBIA, six land-use classes were produced from the Sentinel-2 image. The accuracy of the categorization was compared in the classification report using the ground truth data. An overall degree of precision of 88.18% was succeeded, with a Kappa coefficient of 0.86. As illustrated in Figure 8a and Table 6, agroforestry was the leading LULC type, making up 44.54% of the research site followed by farmland (41.34%), forest (7.33%), settlement (4.85%), wetland (1.22) and grassland (0.72%). From these LULC types, agroforestry was suitable for coffee plantation. Farmland and grassland were reclassified as sub-suitable for coffee farming; and all other LULC types were reclassified as unsuitable (Figure 8d).

##### Distance to Road and River

Euclidian distance to road (Figure 8b) and river (Figure 8c) were derived and reclassified based on their impact for coffee plantation. The potential decreases as distance increases from road (Figure 8e), and river (Figure 8f).

### 2.7. Analytic Hierarchy Process

The analytical hierarchy process (AHP) is the supreme well-known approach for weighing and rating locations in order to select a suitable site. AHP is a decision-constructing assistance technique that allows an alternative solution to be selected from a list of possibilities [74]. The dynamic model is specifically designed to tackle the real problem of the research area, which is selecting a suitable location from a range of options. After the datasets were reclassified, the AHP generated a matrix of pairwise contrasts so that every criterion could be contrasted with the others [75]. This method establishes a hierarchy of criteria for making decisions by comparing each pair of objects represented as a matrix. Analyzing ecological appropriateness requires calculating and evaluating the influence and relative relevance of numerous variables [22]. Decision-making using multiple criteria is an indispensable technique for ecological appropriateness localization, and it has a remarkable structure and accuracy of decision-making. Pair comparison simplifies the computing process. The method has been widely applied in conjunction with GIS in crop ecological suitability studies [76]. In this study, AHP verified the relative importance of metrological, edaphic, physiographic, and socioeconomic factors. Using AHP and GIS together, three categories of suitable, sub-suitable, and unsuitable sites were identified. Thus, the crop’s ecological adaptability was verified.

To ascertain each criterion layer’s relative value, reclassification and ranking were carried out [13]. As a result, the conclusion was influenced by the reclassified criteria layers through a quantitative rank. The weight assignment for the criterion layers was performed using the pairwise comparison approach. The researcher’s expertise and interviews with different experts were used to create the pairwise comparison matrices [13]. Nigussie et al. [13] and Maulana and Kana [77] state that the AHP approach for determining potentiality consists of four core phases. The first stage is to choose the criteria and sub-criteria. The next phase is to establish a hierarchical arrangement. Creating a matrix of pairwise comparisons depending on the relative weights of each criterion is the third stage. Completing the matrix calculation and verifying its consistency is the fourth step. A nine-point rating system was applied, following [78,79] guidelines, where 1 indicated that the compared criteria had equal weight and 9 indicated that one criterion was highly important in contrast to the other.

The upper and lower criteria of the hierarchical decision model were ranked using Sat’s nine-point rating system. The determination of the scale for every element involved examining real-world problems and accounting for the ways in which each criterion influenced the suitability of the coffee land. At each level, pairs of matrix elements were compared to determine how important each element is to the component of the subsequent higher level. Pairwise comparison square matrixes were created by starting at the top of the hierarchy and working down so that each of the matrix’s elements is positive as given in Equation (1).

(1) $$A=[aij]=\left[\begin{array}{c} a11\;a12\;\ldots \;a1n\\ a21\;a22\;\ldots \;a2n\\ \ldots \;\ldots \;\ldots \\ an1\;an2\;\ldots \;ann \end{array}\right]$$

Such that [aij] > 0.

The square matrix’s diagonal components are always 1 due to the uniform weight of the factors. As a result, the factors should be compared to each other in order to create the upper and lower triangular matrices. In general, the reciprocity property of a matrix is represented in mathematical terms $\frac{n(n-1)}{2}$ for a pairwise comparison matrix with n elements. Using Equation (2), the lower triangular matrix is completed.

(2) $$aji=\frac{1}{aij}$$

Next, each column’s components are added together, and each column’s section is divided by the total of its component columns to obtain the normalized relative weight [78], using Equation (3).

(3) $$W*=\frac{aij}{\sum_{i=1}^{n}aij}\;(3)$$

W∗ is the normalized weight and n is the number of items in a column.

Subsequently, averaging the normalized relative weights in a row for each class within a factor associated with higher hierarchy yields the weights of the pairwise matrix as given in Equation (4).

(4) $$W=\frac{\sum_{j=1}^{n}\left(W*\right)ij}{n}$$

for all i=1,2…n. 

Based on [78,79], the consistency ratio (CR) was evaluated using the ratio of consistency index (CI) and random consistency index (RI).

The overall weight of each criterion layer was calculated by multiplying the weight of a factor in the lower level by the weights of the elements in the upper level. For example, the overall weight of mean annual rainfall was computed by the product of the weight of climatological and weight of mean annual rainfall.

Identification of potential areas for coffee plantation based on Criteria Weights.

According to [13], the reclassified factors are merged by utilizing a weight to each continued by a summation of the results. For the current study, potential areas for coffee plantations were derived based on reclassified factors and the weight of each factor.

PACP=∑wixi,

where *PACP* is Potential areas for the coffee plantation, wi is the weight of the factor i and xi is the criterion score of the factor i.

## 3. Results

### 3.1. Identified Coffee Spatial Coverage

**Figure 9:**
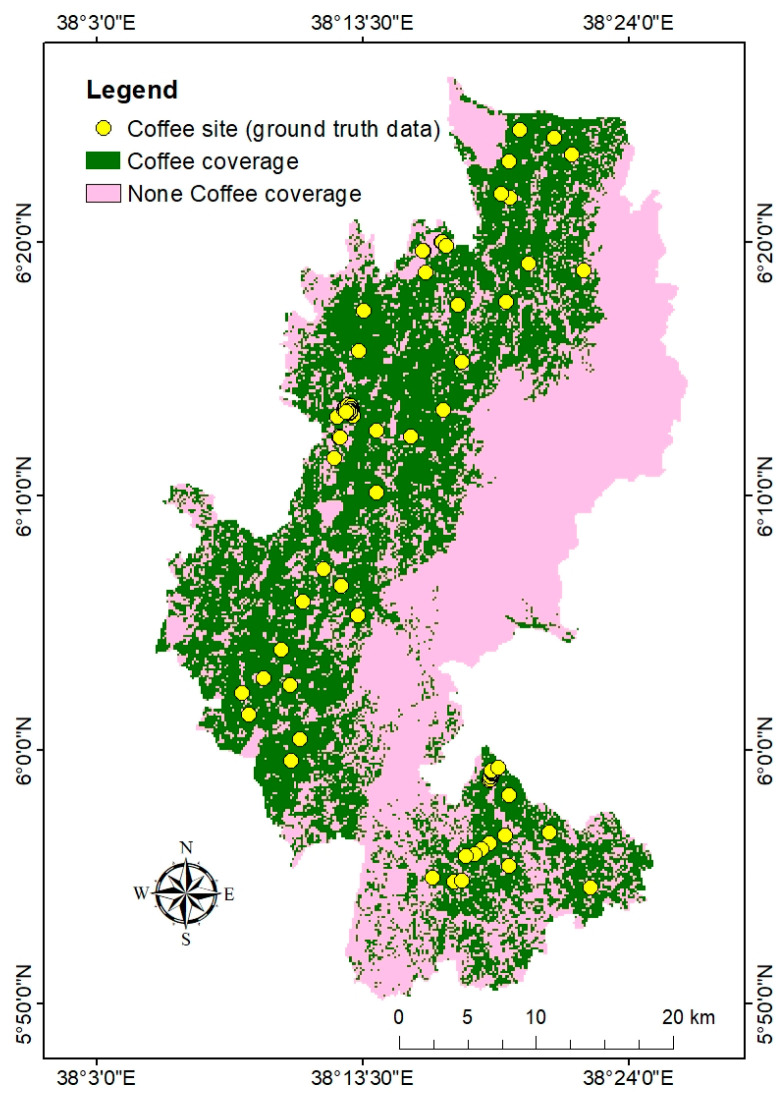
Current potential coffee coverage areas in Gedeo zone.

The current estimated coffee coverage from Sentinel-1 data in the Gedeo zone is 583 km^2^ (Figure 9). Most of the northwestern and southern parts of the Gedeo zone are covered by coffee vegetation. Coffee plantations largely cover the Weyina Dega agro-ecological zone in the research area. Dilla Zuria, Yirgacheffe, and Gedeb are the main known coffee coverage areas. In the rugged topography of Gedeo zone, coffee did not cover the eastern Dega agro-ecological zone. However, in the southern Dega part, field observations and actual mapped coffee have shown the existence of some coffee farms. The collected ground truth data from coffee farm sites were correlated with mapped coffee coverage.

### 3.2. AHP-Based Derived Weights for Coffee Suitability

The pairwise comparison matrix of main factors (climatology, edaphic, physiographic and socioeconomic) is illustrated in Table 7A. This is because not all main factors and sub-factors can equally influence the selection of potential sites for coffee growing. The weights of primary factors (Table 7C) were computed using a normalized matrix obtained in Table 7B. Based on weighted sum and priority, λmax was 4.08. The computed CI results are 0.0267. Then, the CR was 0.029. Pairwise analysis of the primary criteria in the upper hierarchy (Table 7A) indicates that climatology is more important than the other three primary components.

Upon further analysis of the pairwise comparisons of the primary criteria (Table 7A), it is evident that climatology is the most significant factor, with a weight of 0.66 (Table 7C). This high weight is justified, as climatic conditions play a critical role in Ethiopia’s coffee belts. The next most important factor is the edaphic component, with a weight of 0.16, which is essential for coffee growth and development, particularly in terms of root extension and soil drainage. The physiographic and socioeconomic factors were assigned weights of 0.15 and 0.09, respectively, and their influence on coffee-growing suitability is also discussed in greater depth.

#### Metrological, Edaphic, Physiographic, and Socioeconomic Sub-Factors

The comparison matrix, normalized matrix, and priority weight of the meteorological, edaphic, physiographic, and socioeconomic sub-factors are illustrated in Table 8. Table 8A presents the pairwise comparison matrix of metrological sub-factors. Based on the normalized matrix of metrological sub-factors (Table 8B), priority weights of metrological sub-factors were derived. As shown in Table 8C, average annual temperature is the primary factor with a weight of 0.39 followed by mean annual rainfall (0.27). The derived weight for the annual mean maximum and minimum temperature was 0.21 and 0.13, respectively. Based on the given pairwise comparison matrix of edaphic sub-factors (Table 8D), the normalized matrix (Table 8E) and priority weight of edaphic sub-factors (Table 8F) were computed. Since the growth of coffee is controlled by soil pH and texture, soil pH is the dominant factor, followed by soil texture in the edaphic sub-criteria and their weights were 0.37 and 0.28, respectively. The weights computed for CEC and SOM were 0.2 and 0.15, which contributed less weight to identifying potential coffee plantation areas. In the third main factor, pairwise comparison matrixes of physiographic sub-factors were presented in Table 8G. After computing normalized matrix (Table 8H), the weight of physiographic sub-factors were derived (Table 8I). In the physiographic sub-criteria, elevation contributed the dominant factor followed by slope and aspect, with their computed weights of 0.65, 0.21, and 0.14, respectively. The fourth pairwise comparison matrix is the socioeconomic sub-factors, presented in Table 8J. The priority weights of socioeconomic sub-factors were calculated (Table 8L) based on normalized matrix of socioeconomic sub-factors (Table 8K). In the socioeconomic factors, LULC was the dominant factor weight (0.6), followed by distance to roads (0.23) and rivers (0.17).

### 3.3. Overall Weight of Thematic Layers

The final weight of each thematic factor was computed by multiplying the main factor weight with each sub-factor weight (Table 9). The highest weight in the main factors contributed to having the highest final weight for the associated sub-criteria. Climate, as the primary factor, has the highest weight of 0.6, which was used to compute the final weights of its sub-factors. For example, the final weight of average annual temperature (0.234) was calculated by multiplying the climate weight (0.6) by the weight of average annual temperature (0.39). In addition to presenting the data, we have analyzed what the weights imply for coffee suitability. For instance, the high weight of climate factors, particularly average annual temperature and mean annual rainfall, emphasizes the importance of climatic conditions in determining coffee plantation suitability. These factors dominate because they directly affect the growing conditions essential for coffee production in the region. Other factors like elevation and soil pH also play a critical role, as they influence the microclimate and soil properties, which are essential for coffee development. As shown in Table 9, the CR value for all main factors and sub-factors is less than 0.1, indicating that the AHP matrix used for this study is reasonably consistent. This consistency allows us to confidently proceed with the decision-making process to identify potential areas for coffee plantations. The final computed weights reveal that the most dominant factors for coffee suitability are average annual temperature, mean annual rainfall, and elevation, followed by other factors such as soil pH, LULC, and distance to roads and rivers.

### 3.4. Identified Coffee Plantation Potential Land Suitability

**Figure 10:**
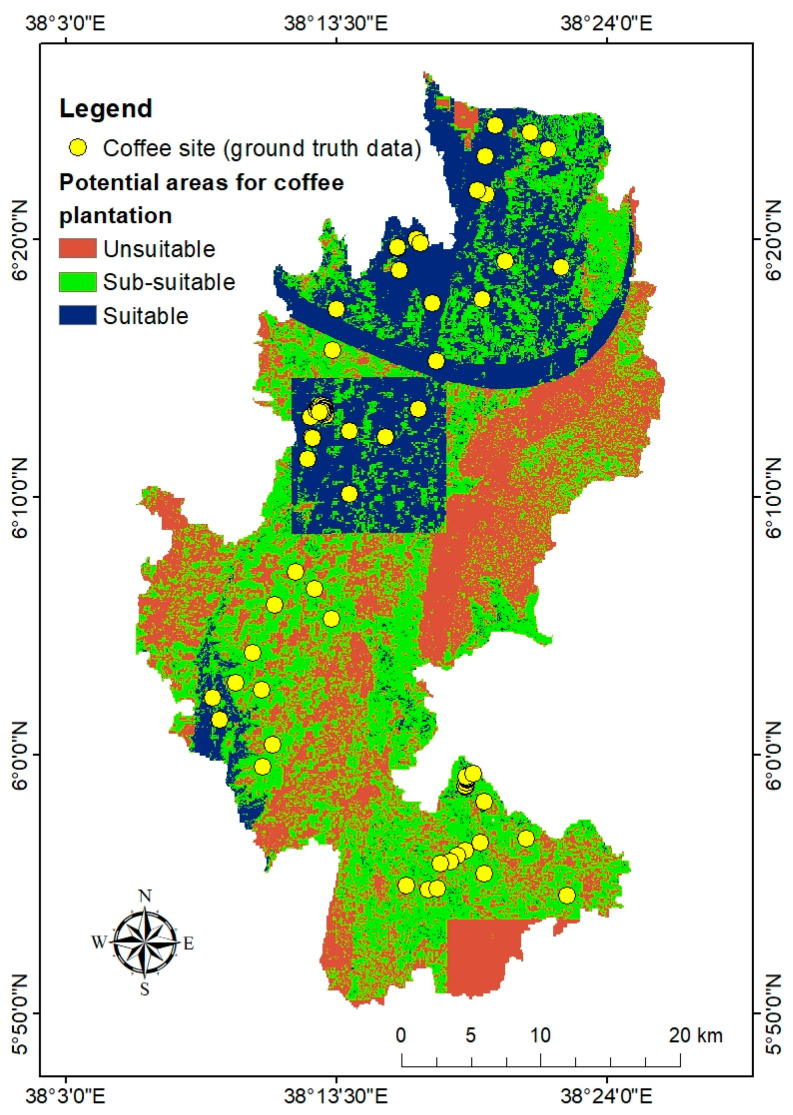
Identified potential areas for coffee plantation.

**Figure 11:**
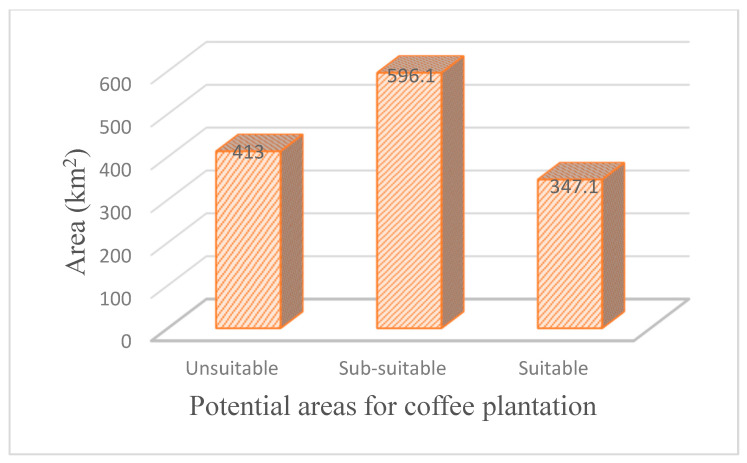
Area coverage of identified potential coffee plantation.

Coffee potential locations within the research area were identified using the fourteen reclassified thematic layers and their respective percentage of influence. The identified coffee potential areas (Figure 10) showed that the suitable areas for growing coffee were located in the northwestern part of the study area. This followed a pattern as many input conditions proved appropriate, in which the average annual temperature and mean annual rainfall are the primary factors, followed by annual mean maximum temperature, elevation, annual mean minimum temperature, soil pH, LULC, soil texture, CEC, slope, SOM, aspect, distance to roads, and distance to water, respectively. Most sub-suitable areas were found in the southern and central south-north parts of the study area. It was also observed that unsuitable areas existed in the eastern and southern Dega parts of the research site. The suitable and sub-suitable coffee-growing potentials accounted for 25.6% and 44% of the total study area, respectively (Figure 10, Table 10). The unsuitable districts accounted for 30.4% of the overall area. The collected coffee farm data were overlaid on the identified coffee potential area (Figure 11) and showed that most of them align with suitable, and some with sub-suitable potential. This demonstrated that the method was convincing for identifying potential coffee plantations.

## 4. Discussion

Most of the time, qualitative and quantitative data have been used to determine the coverage of Ethiopian coffee instead of mapping spatially where the exact coffee forest is situated [80]. Mapping coffee plantations using satellite imagery is challenging due to spectral similarities with other plantations. Thus, the classification scheme for mapping coffee plantations requires a proper understanding of the data used in the field. Sentinel-1 is crucial to map shaded coffee coverage, due to its ability to penetrate shaded agroforestry coverage. In the current study, using field data and sentinel-1, the mapped coffee coverage is 583 km^2^ mainly situated in the northern and southern regions. Coffee experts and farmers stated that the climate change in the site area influenced the adaptation of coffee plantations. In complicated topographies with diversified landscapes and somewhat higher elevations, coffee is primarily produced on small farms [81,82]. In rugged topography of Gedeo zone, the identified coffee coverage is mostly located in elevation ranges of 1500 to 2300 m above mean sea level. Improved production and livelihoods for smallholder farmers depend on the identification of suitable agro-ecological zones and an awareness of climate-related challenges [83]. Finding suitable sites for agricultural practices is vital to increasing food and cash crop production. To promote successful and productive planning and land-use management procedures, land can be divided according to its suitability for a particular purpose [84]. Using 14 thematic layers from climatic, edaphic, physiographic, and socioeconomic elements, this study has analyzed and identified viable areas for coffee plantations. Coffee’s development and growth are largely influenced by temperature [22]. In Ethiopia, the annual average temperature has increased by 0.32 °C every decade, while rainfall has declined by 5% to 20% [21,60]. As a result, the frequency of droughts has increased, making less land suitable for growing Arabica coffee.

The current study considers the 30-year temperature and rainfall climatic layers as the dominant factors for coffee plantation land suitability. The mean annual rainfall of the Gedeo zone ranges from 1093.02 to 1667 mm, of which 1600–1667 mm and 1100–1600 mm contributed to suitable and sub-suitable coffee plantations, respectively. The average annual temperature ranges from 16.95 to 20.35 °C, with 18–20.35 °C and 15–18 °C thresholds subsidized for suitable and sub-suitable potential for coffee plantations. Elevation in the physiographic elements had the highest impact on the ecological adaptability of coffee plantation [22]. Similarly, this study also used elevation as the greatest influence on the physiographic factor. The elevation of the Gedeo zone extends from 1329 to 3088 m above mean sea level. In the study area, suitable altitude ranges are 1400–1800 m, and sub-suitable elevation ranges are 1329–1400 m and 1800–2500 m. The unsuitable altitude ranges for coffee farming are 2500–3088 m.

At a 250 m spatial resolution and a 0–30 cm depth interval, spatial predictions of the macro- and micronutrient levels of soil were made for Sub-Saharan Africa [41]. The optimum pH for Coffea arabica is 5–6.5 [22]. Soil pH is the most influential layer among the edaphic factors. Mostly, the soil pH in the Gedeo zone is acidic, ranging from 0 to 7. Suitable pH for coffee plantations ranges from 5 to 6.5, and sub-suitable pH ranges from 4.5 to 5 and 6.5 to 7. Sandy loam and sandy clay loam soil textures are the most suitable for coffee plantations [62]. In the current study, loam and sandy loam contributed to suitable and sub-suitable potential coffee plantations. In the socioeconomic sub-factors, LULC is the dominant factor contributing to the identified potential area for coffee plantations. Agroforestry and farmland are suitable and sub-suitable for potential coffee growing. As the distance from roads and rivers increases, the potential for coffee growing decreases. The AHP-GIS approach has been extensively utilized in diverse investigations, such as eco-environmental relationships, agricultural land categorization, groundwater potential mapping, risk management analysis, regional evaluation, and ecological appropriateness studies of various crops [13,57,85]. For the present research, the AHP model was used to derive the relative weight of each of the 14 thematic layers. A weight was assigned to each of the reclassified criteria, and the results were then combined, yielding coffee plantation potential land suitability, which was classified as unsuitable (30.4%), sub-suitable (44%), and suitable (25.6%). Although mapping spatial coffee coverage is challenging, coffee production has a significant role in national politics and the economy of coffee-growing [86]. The Ethiopian government recently plans to invest billions of Birr for managing coffee plantations. In the current study site, the government established institute of coffee center at Yirgachefe. As primary research, this study has a significant role in showing the actual coffee coverage and prioritizing potential areas for coffee plantations in diverse topography and heterogeneous agroforestry region of Gedeo zone. Comparisons between the model findings of coffee suitability maps and the locations of currently operating coffee farms were necessary for validation [84]. Similar to Rono and Mundia [84], this study’s result of the identified coffee plantation potential land suitability was validated by using field coffee farm sites. The collected coffee farm sites were overlaid on identified coffee potential areas, and all of them matched with identified sub-suitable and suitable potential areas.

Previous research, such as [32], used Landsat images with supervised classification for LULC derivation and coffee plantation mapping, resulting in lower accuracy than the current study’s results and verifications. Similarly, Mukashema et al. [87] used high-resolution aerial orthophotos for coffee mapping, producing quality results based on ground truth data verification. Studies like [32,33] have conducted comparative analyses for coffee mapping and LULC classification techniques, showing that high-resolution images like Sentinel-1 and Sentinel-2, as used in our study, provide more effective results for coffee mapping and LULC studies. These findings are in line with the results of this study. Consistent with the findings of [22,54,55,56], which worked in different study areas, the coffee suitability results of this study are predominantly located in the Weyina Dega region, where optimum rainfall and temperature conditions for coffee cultivation are present. This reinforces the reliability of the methods employed in this study and demonstrates that they are acceptable for broader application.

Machine learning techniques, such as the random forest algorithm, are crucial for understanding how various influencing factors relate at different spatial scales [88]. However, this study did not employ machine learning, as the verification using ground truth data from coffee farms demonstrated that the methodology and outcomes corresponded well with the actual conditions. Future research should build on this approach by incorporating machine learning methods to improve the selection of suitable areas for coffee cultivation. One limitation of this study is the lack of validation for edaphological sub-factors through laboratory tests. Furthermore, constraints related to the coffee market and policy orientations were not considered. Thus, we recommend that future studies build upon the approach used in this study by incorporating machine learning methods, laboratory validations of edaphological factors, and accounting for market and policy constraints to enhance the selection of suitable areas for coffee cultivation.

## 5. Conclusions

This study aimed to map coffee spatial coverage and identify land suitability for coffee plantations in the diverse topography and heterogeneous agroforestry region of the Gedeo zone. The challenge of mapping coffee farms stems from the spectral similarity between coffee plantations and other types of farmland, making it difficult to differentiate coffee areas using satellite imagery alone. To address this, we integrated multispectral (Sentinel-2) and SAR (Sentinel-1) remote sensing data with GIS and the Analytical Hierarchy Process (AHP). This combination proved to be an effective, practical, and economical approach for mapping shaded coffee areas and identifying potential land suitable for coffee plantations. In this study, the estimated coffee coverage was 583 km^2^. Fourteen thematic factors were used to derive land suitability for coffee plantations. The AHP method was particularly useful for assessing the relative importance of these factors. The consistency ratio (CR) of 0.029 was within the acceptable range, reinforcing the reliability of the analysis. The weight comparison of thematic factors indicated that average annual temperature, mean annual rainfall, annual maximum temperature, elevation, annual minimum temperature, soil pH, and LULC were the most significant factors influencing potential coffee plantation suitability. Secondary factors included soil texture, CEC, slope, SOM, aspect, and proximity to roads and rivers.

Using the weighted linear combination based on the reclassified values of the 14 thematic layers, we identified the suitable areas for coffee cultivation. The results show that 30.4% of the area is unsuitable, 44% is sub-suitable, and 25.6% is suitable for coffee cultivation. Most of the suitable and sub-suitable areas are located in the Woina Dega agro-ecological zone, where favorable climatic conditions and agroforestry LULC types prevail. In contrast, the eastern Dega part of the Gedeo zone (Rape and Bule district) is unsuitable for coffee cultivation.

Ground truth data from coffee farms were used to verify the results, confirming that the methodology and suitability analysis closely matched the actual conditions on the ground. This study provides detailed spatial information that can guide farmers, agricultural extension workers, and government officials in selecting optimal locations for coffee production. The findings are particularly valuable for farmers, investors, researchers, and NGOs interested in cultivating coffee in the Gedeo zone. Focus should be directed toward the suitable and sub-suitable areas identified in the study. Finally, since mapping existing coffee coverage is crucial for investors and exporters, shaded coffee areas should continue to be mapped using active Sentinel-1 data. Future studies should incorporate additional restrictions, such as market conditions, policy orientations, and laboratory validation of soil characteristics, to refine the process of identifying suitable areas for coffee plantations.

## Figures and Tables

**Table 1:** Geospatial data used.

Major Data	Source	Resolution	Purpose
Sentinel-2	Copernicus	10 m	To generate LU/LC
Sentinel 1	Copernicus hub	10 m	To map coffee coverage
ALOSE DEM	Alaska facility	12.5 m, resampled to 10 m	To generate elevation, terrain slope, terrain aspect, and distance to water network
Rainfall (CHIRPS)	Earth Resources Observation and Science Center	5 km (resampled to 10 m)	To derive mean annual rainfall
Temperature	Ethiopian National Metrological Agency	Interpolated and resampled to 10 m	To derive annual mean maximum temperature, annual mean minimum temperature, and average annual temperature
Soil texture	Ethiopian Ministry of Agriculture	250 m (resampled to 10 m)	To get soil texture map
Soil pH, Organic matter, Cation exchange capacity	Africa Soil Information Services https://soilgrids.org/ (accessed on 1 September 2024)	250 m (resampled to 10 m)	To get Edaphalogical sub-factors (soil pH, Organic matter, and Cation exchange capacity)
Topographic map	Ethiopian Space Technology and Geospatial Institute	1:50,000	To obtain distance to road network, and for Field guideline

**Table 2:** Agro-ecological classification [49].

Elevation (Meters above Mean Sea Level)	Agro Ecology Class
<500	Berha
500–1500	Kolla
1500–2300	Weyina Dega
2300–3200	Dega
3200–3700	Wurch
>3700	High Wurch

**Table 3:** Sub-criteria suitability thresholds (requirements) for coffee growing, adapted from [22,54,55,56,57,58,59,60].

Sub-Criteria/Criteria	Suitable (3)	Sub-Suitable (2)	Unsuitable (1)
Climatological			
Mean annual rainfall (mm)	1600–1800	1100–1600; 1800–2000	<1100; >2000
Average annual temperature (°C)	18–23	15–18; 23–26	>26; <15
Annual mean min temperature (°C)	>18	10–18	<10
Annual mean max temperature (°C)	<25	25–30	>30
Edaphological			
Texture (texture class)	L, SCL,SiCL	CL, SL, SC, SiL, SiC	S, C, Si, LS
pH	5–6.5	4.5–5; 6.5–7.5	<4.5; >7.5
SOM (%)	>3	2–3	<2
CEC (cmol+/Kg)	>25	15–25	<15
Physiographic			
Elevation (m a.s.l.)	1400–1800	900–1400; 1800–2500	<900; >2500
Terrain slope (%)	0–12	12–25	>25
Terrain aspect	N, NE, NW	E, W	S, SW, SE
Socioeconomic			
LULC	Agroforestry	Farmland, grassland	Forest, settlement, wetland
Distance to water network (km)	0–1	1–5	>5
Distance to road network (km)	0–2	2–5	>5

**Table 4:** Soil texture and study area coverage.

Soil Texture Type	Area (km^2^)
Clay	71.86
Loam	382.87
Sandy Loam	901.4

**Table 5:** Annual mean minimum and maximum temperature.

Metrological Station	Elevation	Lat	Long	Annual Mean Min Temp (°C)	Annual Mean Max Temp (°C)
Dilla	1515	6.380556	38.30694	12.7	28
Fiseha Genet	2240	6.0667	38.18333	12.1	22
Yirga Chefe	1856	6.150667	38.202	10.4	24.5
Hagere Selam	2809	6.49	38.52	7	19.2
Gedebe	2245	5.905667	38.2395	11.9	22.02

**Table 6:** LULC areas for the reference year 2021 of the study areas.

LULC Types	2021	
	Area (sq km)	%
Farmland	560.6	41.34
Agroforestry	604.1	44.54
Forest	99.4	7.33
Grassland	9.8	0.72
Settlement	65.8	4.85
Wetland	16.5	1.22
Total	1356.2	100

**Table 7:** Comparison matrix, normalized matrix, and priorities of main factors.

**A. Pairwise comparison matrix of main factors**						
**Coffee Potential**	**Climatology**	**Edaphic**	**Physiographic**	**Socioeconomic**		
Climatology	1	5	4	5		
Edaphic	1/5	1	3/2	3/2		
Physiographic	1/4	2/3	1	2		
Socioeconomic	1/5	2/3	1/2	1		
Sum	1.65	7.333	7	9.5		
**B. Normalized matrix of main factors**						
**Coffee Potential**	**Climatology**	**Edaphic**	**Physiographic**	**Socioeconomic**	**Sum**	
Climatology	0.606	0.682	0.571	0.526	2.385	
Edaphic	0.121	0.136	0.214	0.158	0.629	
Physiographic	0.151	0.091	0.143	0.21	0.595	
Socioeconomic	0.121	0.091	0.071	0.105	0.388	
**C. Priorities (Weights) of main factors**						
**Coffee Potential**	**Climatology**	**Edaphic**	**Physiographic**	**Socioeconomic**	**Sum**	**Weight**
Climatology	0.606	0.682	0.571	0.526	2.385	0.60
Edaphic	0.121	0.136	0.214	0.158	0.629	0.16
Physiographic	0.151	0.091	0.143	0.21	0.595	0.15
Socioeconomic	0.121	0.091	0.071	0.105	0.388	0.09

**Table 8:** Comparison matrix, normalized matrix, and priority weight of the metrological, edaphic, physiographic, and socioeconomic sub-factors.

**A. Pairwise comparison matrix of metrological sub-factors**						
**Coffee Potential**	**Mean Annual Rainfall**	**Average Annual Temp**	**Annual Mean Min Temp**	**Annual Mean Max Temp**		
Mean annual rainfall	1	2/3	2	3/2		
Average annual temp	3/2	1	3	2		
Annual mean min temp	1/2	1/3	1	2/3		
Annual mean max temp	2/3	2/3	3/2	1		
Sum	3.667	2.667	7.5	5.167		
**B. Normalized matrix of metrological sub-factors**						
**Coffee Potential**	**Mean annual rainfall**	**Average annual temp**	**Annual mean min temp**	**Annual mean max temp**	**Sum**	
Mean annual rainfall	0.273	0.25	0.266	0.29	1.079	
Average annual temp	0.409	0.375	0.4	0.387	1.571	
Annual mean min temp	0.136	0.125	0.133	0.13	0.524	
Annual mean max temp	0.182	0.25	0.2	0.193	0.825	
**C. Priority weight of metrological sub-factors**						
**Coffee Potential**	**Mean annual rainfall**	**Average annual temp**	**Annual mean min temp**	**Annual mean max temp**	**Sum**	**Weight**
Mean annual rainfall	0.273	0.25	0.266	0.29	1.079	0.27
Average annual temp	0.409	0.375	0.4	0.387	1.571	0.39
Annual mean min temp	0.136	0.125	0.133	0.13	0.524	0.13
Annual mean max temp	0.182	0.25	0.2	0.193	0.825	0.21
**D. Pairwise comparison matrix of edaphic sub-factors**						
**Coffee Potential**	**pH**	**texture**	**CEC**	**SOM**		
pH	1	1.5	2	2		
texture	0.667	1	1.5	2		
CEC	0.5	0.667	1	1.5		
SOM	0.5	0.5	0.667	1		
Sum	2.667	3.667	5.167	6.5		
**E. Normalized matrix of edaphic sub-factors**						
Coffee Potential	pH	texture	CEC	SOM	Sum	
pH	0.375	0.409	0.387	0.308	1.479	
texture	0.25	0.273	0.29	0.308	1.121	
CEC	0.188	0.182	0.193	0.231	0.794	
SOM	0.188	0.136	0.129	0.154	0.607	
**F. Priority weight of edaphic sub-factors**						
Coffee Potential	pH	texture	CEC	SOM	Sum	Weight
pH	0.375	0.409	0.387	0.308	1.479	0.37
texture	0.25	0.273	0.29	0.308	1.121	0.28
CEC	0.188	0.182	0.193	0.231	0.794	0.2
SOM	0.188	0.136	0.129	0.154	0.607	0.15
**G. Pairwise comparison matrix of physiographic sub-factors**						
Coffee Potential	Elevation	Slope	Aspect			
Elevation	1	3	5			
Slope	1/3	1	3/2			
Aspect	1/5	2/3	1			
Sum	1.533	4.667	7.5			
**H. Normalized matrix of physiographic sub-factors**						
Coffee Potential	Elevation	Slope	Aspect	Sum		
Elevation	0.652	0.643	0.667	1.962		
Slope	0.217	0.214	0.2	0.631		
Aspect	0.13	0.143	0.133	0.406		
**I. Priority weight of physiographic sub-factors**						
	Elevation	Slope	Aspect	Sum	Weight	
Elevation	0.652	0.643	0.667	1.962	0.65	
Slope	0.217	0.214	0.2	0.631	0.21	
Aspect	0.13	0.143	0.133	0.406	0.14	
**J. Pairwise comparison matrix of socioeconomic sub-factors**						
Coffee Potential	LULC	Distance to road	Distance to river			
LULC	1	3	3			
Distance to road	1/3/	1	3/2			
Distance to river	1/3/	2/3/	1			
Sum	1.667	4.667	5.5			
**K. Normalized matrix of socioeconomic sub-factors**						
Coffee Potential	LULC	Distance to road	Distance to river	Sum		
LULC	0.6	0.643	0.545	1.788		
Distance to road	0.2	0.214	0.273	0.687		
Distance to river	0.2	0.143	0.182	0.525		
**L. Priority weight of socioeconomic sub-factors**						
	LULC	Distance to road	Distance to river	Sum	Weight	
LULC	0.6	0.643	0.545	1.788	0.6	
Distance to road	0.2	0.214	0.273	0.687	0.23	
Distance to river	0.2	0.143	0.182	0.525	0.17	

**Table 9:** Final weight of thematic factors.

Main Factor	Weight	CR	Factor	Weight	CR	Final Weight	%
Climate	0.6	0.029	Mean annual rainfall	0.27	0.03	0.162	16.2
			Average annual temperature	0.39	0.03	0.234	23.4
			Annual mean min temperature	0.13	0.03	0.078	7.8
			Annual mean max temperature	0.21	0.03	0.126	12.6
Edaphic	0.16	0.029	pH	0.37	0.008	0.0592	5.92
			Texture	0.28	0.008	0.0448	4.48
			CEC	0.2	0.008	0.032	3.2
			SOM	0.15	0.008	0.024	2.4
Physiographic	0.15	0.029	Elevation	0.65	0.001	0.0975	9.75
			Slope	0.21	0.001	0.0315	3.15
			Aspect	0.14	0.001	0.021	2.1
Socioeconomic	0.09	0.029	LULC	0.6	0.01	0.054	5.4
			Distance to road	0.23	0.01	0.0207	2.07
			Distance to river	0.17	0.01	0.0153	1.53
Total						1	100

**Table 10:** Area coverage of identified potential coffee plantation.

Potential Areas for Coffee	Area (km^2^)	Area (%)
Unsuitable	413	30.4
Sub-suitable	596.1	44
Suitable	347.1	25.6
Total	1356.2	100

## Data Availability

Derived data supporting the findings of this study are available from the first author on request.
